# Compression and Superficial Varicosities Outperform Gonadal Vein Diameter in Differentiating Symptomatic from Asymptomatic Pelvic Venous Disorders: A Case–Control Study

**DOI:** 10.1007/s00270-026-04493-5

**Published:** 2026-06-07

**Authors:** Estefania Gonzales, Victoria Nguyen, Victoria Risner, Lourens du Pisanie, Nicole Keefe, Priya Mody, Marueen P. Kohi, Gloria Salazar

**Affiliations:** 1 Department of Radiology, University of North Carolina School of Medicine, 2000 Old Clinic, CB #7510, Chapel Hill, NC USA; 2 Division of Vascular and Interventional Radiology, Department of Radiology, University of North Carolina School of Medicine, Chapel Hill, NC USA

**Keywords:** Pelvic venous disorders, Pelvic congestion syndrome, Chronic pelvic pain, Gonadal (ovarian) vein, Venous outflow obstruction, Renal vein compression, Iliac vein compression, Varicosities, May-Thurner syndrome, Nutcracker syndrome, Lower-extremity varices

## Abstract

**Purpose:**

To assess whether gonadal vein (GV) diameter associates with pelvic venous disorder (PeVD)-consistent symptoms in women with pelvic varices, compared with proximal venous outflow obstruction (VOO; iliac/left renal vein stenosis/obstruction) and superficial varicosities.

**Materials and Methods:**

This retrospective, case–control study identified patients with abdominopelvic imaging (2013–2024) showing parauterine/pelvic varices > 5 mm and documentation within ± 12 months for symptom adjudication. GV diameter was measured on venous-phase computed tomography/magnetic resonance imaging by two readers, or from ultrasound when unavailable. Proximal VOO was ascertained by highest-confidence available testing and evaluated with logistic regression.

**Results:**

Of 200 patients (mean age, 52.4 ± 17.0 years), 84 (42.0%) were symptomatic and 116 (58.0%) asymptomatic. Mean GV diameter did not differ by symptom status (8.89 ± 2.29 vs 9.43 ± 2.50 mm; *P* = 0.121) and was not associated with symptoms in crude (odds ratio [OR], 0.91 per mm; 95% confidence interval [CI], 0.80–1.03) or adjusted analysis (adjusted OR [aOR], 0.97 per-mm; 95% CI, 0.81–1.14). Proximal VOO was assessable in 189 patients and more common in symptomatic vs asymptomatic patients (32.9% vs 2.6%; *P* < 0.001), as were lower-extremity (LE) varices (44.0% vs 12.9%; *P* < 0.001) and superficial pelvic varicosities (28.6% vs 1.7%; all *P* < 0.001). In the adjusted model, younger age (aOR, 0.50 per 10 years; 95% CI, 0.39–0.65), LE varices (aOR, 9.80; 95% CI, 3.76–25.50), and any proximal VOO (aOR, 10.89; 95% CI, 2.66–44.55) were independently associated with symptomatic status. Discrimination was acceptable (c-statistic, 0.82; 95% CI, 0.76–0.89) and unchanged by GV diameter.

**Conclusion:**

Among women with pelvic varices, GV diameter was not associated with PeVD-consistent symptoms and added no incremental predictive value beyond age, LE varices, and VOO. Comorbid venous disease and outflow obstruction may better distinguish clinically significant PeVD than diameter thresholds alone.

**Graphical Abstract:**

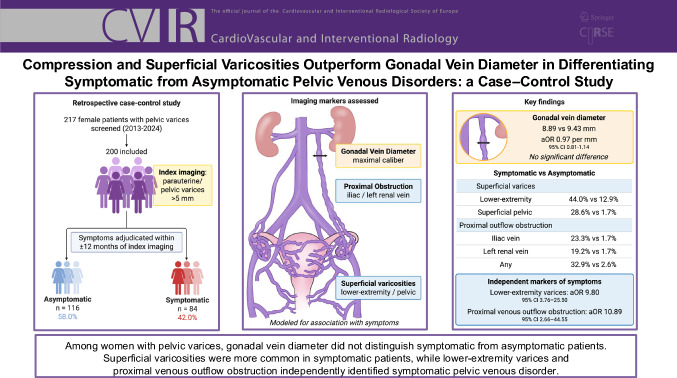

**Supplementary Information:**

The online version contains supplementary material available at 10.1007/s00270-026-04493-5.

## Introduction

Pelvic venous disorders (PeVD) encompass a spectrum of conditions characterized by venous insufficiency within the pelvic vasculature, including pelvic congestion syndrome, ovarian and internal iliac vein reflux, venous outflow obstruction (VOO), and pelvic varicosities [1,2]. Chronic pelvic pain (CPP) is the most common manifestation of PeVD and affects an estimated 15–26% of women aged 18–50; PeVD may account for 30–45% of CPP presentations [3–5]. Additional manifestations include vulvar/perineal and lower-extremity (LE) varicosities, LE pain/swelling related to reflux or VOO, and, less commonly, left flank pain/hematuria in the setting of renal venous compression [2]. Proposed mechanisms include pelvic venous dilation and hypertension with activation of nociceptors and downstream inflammatory signaling [2,6,7].

Diagnostic imaging criteria for PeVD are not standardized, and reported thresholds vary across modalities and studies [2,8]. Diagnosis typically requires correlation of characteristic symptoms (most commonly CPP and dyspareunia) with objective evidence of venous pathology (pelvic varicosities, reflux, VOO, and collateral escape pathways) [9]. Noninvasive imaging, including ultrasound, computed tomography (CT), and magnetic resonance (MR) imaging, is commonly used for initial evaluation, with catheter venography reserved for select cases when hemodynamic assessment or intervention is planned. Dilated pelvic veins alone are frequently reported (e.g., > 5–6 mm), but caliber thresholds are variably applied and may not distinguish symptomatic from asymptomatic patients in the absence of compatible clinical context.

A commonly used radiologic marker for PeVD is gonadal (ovarian) vein dilation, with thresholds ≥ 6 mm often cited [9]. However, prior studies demonstrate inconsistent correlation between gonadal vein (GV) caliber and symptoms: some asymptomatic patients have enlarged GVs, while some symptomatic patients have normal calibers [9–11]. There is a need to better define the relationship between GV dilation and clinically significant PeVD in patients with pelvic varices. Therefore, our study compares GV diameters in symptomatic versus asymptomatic patients with pelvic varices and evaluates whether coexisting venous features (proximal VOO and superficial varicosities) are associated with symptom status.

## Methods

**Fig. 1:**
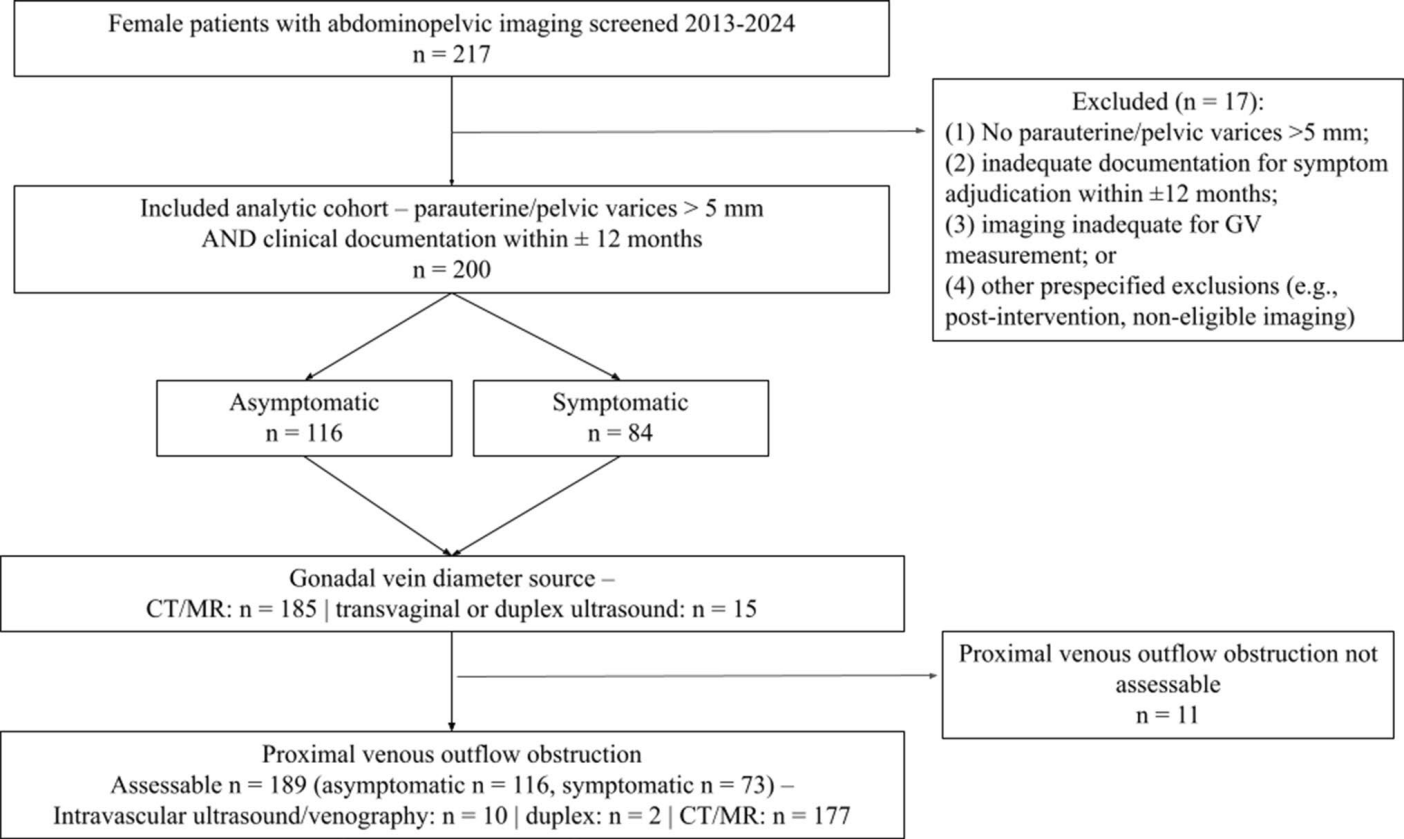
Cohort assembly and variable availability. Female patients with abdominopelvic imaging from 2013–2024 were screened (n = 217); 200 met inclusion criteria (parauterine/pelvic varices > 5 mm and sufficient documentation within ± 12 months of the index imaging date for symptom adjudication). Gonadal vein (GV) diameter was obtained from computed tomography (CT) or magnetic resonance (MR) imaging (n = 185), or from transvaginal or duplex ultrasound when CT/MR was unavailable (n = 15). Proximal venous outflow obstruction (nonthrombotic iliac vein lesion or aortomesenteric left renal vein compression) was assessed by intravascular ultrasound/venography when available, then duplex ultrasound, then CT/MR; proximal venous outflow obstruction was assessable in 189 patients (asymptomatic n = 116, symptomatic n = 73) and not assessable in 11

This institutional review board–approved retrospective, unmatched case–control study (protocol #23-1570) was conducted at a tertiary academic center and used to evaluate factors associated with symptom status rather than population incidence. We queried the Picture Archiving and Communication System (PACS) for female patients with abdominopelvic imaging performed between January 1, 2013, and January 31, 2024 (CT/MR prioritized; ultrasound when CT/MR unavailable), without restriction by imaging indication. Patients were eligible if the index imaging study demonstrated parauterine/pelvic varices > 5 mm and if clinical documentation within ± 12 months of the index study date was sufficient to adjudicate symptom status (Fig. 1). Because the imaging query was indication-neutral, symptomatic and asymptomatic patients were drawn from the same imaged source population.

Patients were excluded if imaging was technically inadequate for GV measurement or if clinical documentation was insufficient to classify symptom status. Patient age was defined at the time of index imaging.

### Index Imaging Review and GV Measurement

**Fig. 2:**
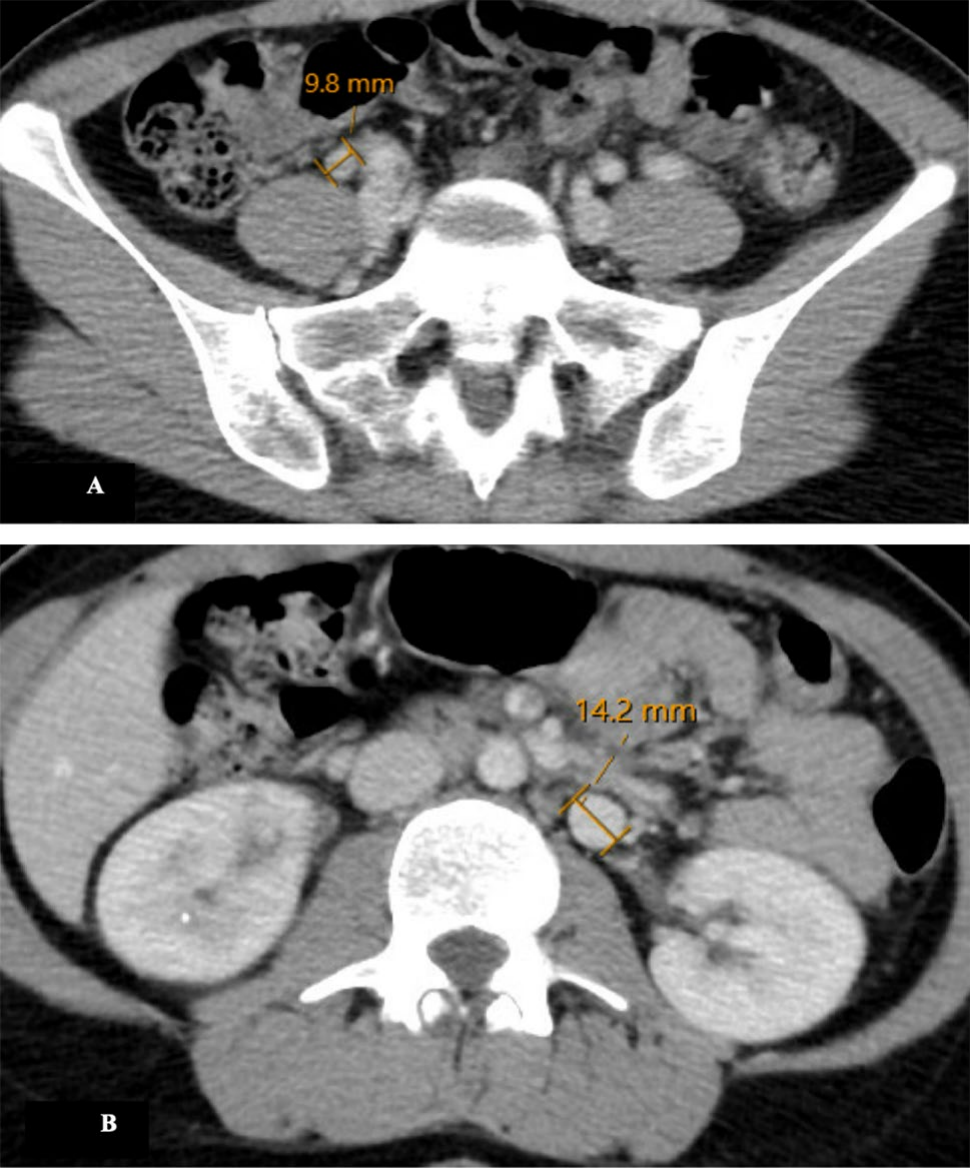
Dilated gonadal veins in asymptomatic patient**.** 22-year-old nulliparous female presenting with 3–4 days of intermittent right-sided abdominal pain and no prior symptoms of chronic pelvic pain. She was found to have pyelonephritis with incidental findings of bilateral gonadal vein dilation. Axial contrast-enhanced computed tomography with measurement calipers (orange) shows a right gonadal vein measuring 9.8 mm (**A**) and left ovarian vein measuring 14.2 mm (**B**)

**Fig. 3:**
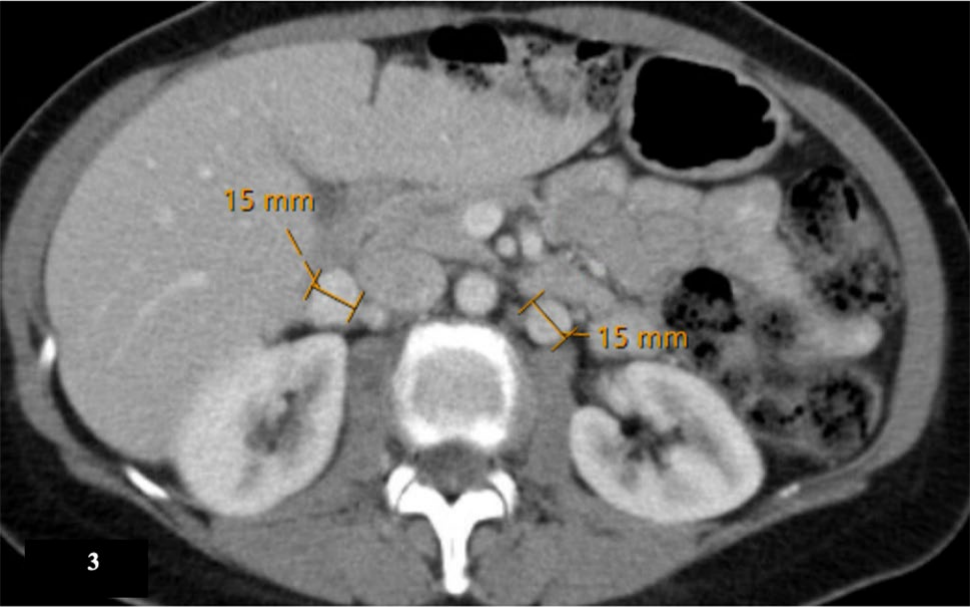
Dilated gonadal veins in patient with pelvic venous disorder. 51-year-old multiparous female presenting with more than 7 months of constant, dull pelvic pain. She later underwent embolization of the left gonadal vein with resolution of chronic pelvic pain symptoms. Axial contrast-enhanced computed tomography with measurement calipers (orange) shows a right gonadal vein measuring 15 mm and left ovarian vein measuring 15 mm

All studies were reviewed on a PACS workstation by a radiologist (> 3 years residency training), blinded to symptom status. The index study was the highest-quality, contrast-enhanced venous-phase CT/MR abdominopelvic examination (including dedicated CT/MR venography when available) obtained prior to any pelvic venous intervention; CT was preferred for diameter measurement, with MR used when venous-phase CT was unavailable or of inferior quality. When CT/MR was unavailable, transvaginal or duplex ultrasound was used as the GV diameter source [12–14]. GV diameter was measured at the point of maximal intraluminal caliber along the imaged vessel course by two independent readers and averaged. Representative examples of GV diameter measurement in asymptomatic and symptomatic patients are shown in Figs. 2 and 3, respectively.

### Proximal VOO Ascertainment

**Fig. 4:**
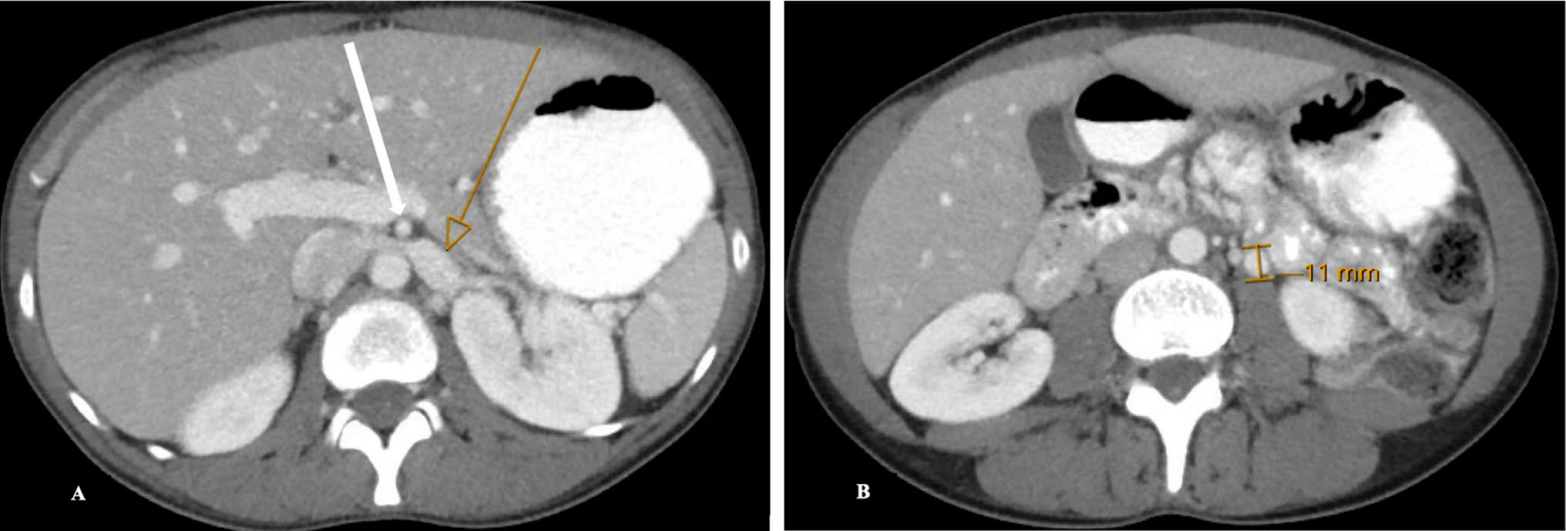
Aortomesenteric left renal vein compression in a patient with pelvic venous disorder. A 35-year-old multiparous female presenting with several months of abdominal pain and nausea. Axial contrast-enhanced computed tomography shows prestenotic dilatation of the left renal vein (orange arrow, Fig. 4A) with focal aortomesenteric narrowing between the superior mesenteric artery (white arrow, **A**) and aorta. The same imaging study shows a dilated left ovarian vein measuring 11 mm (**B**)

**Fig. 5:**
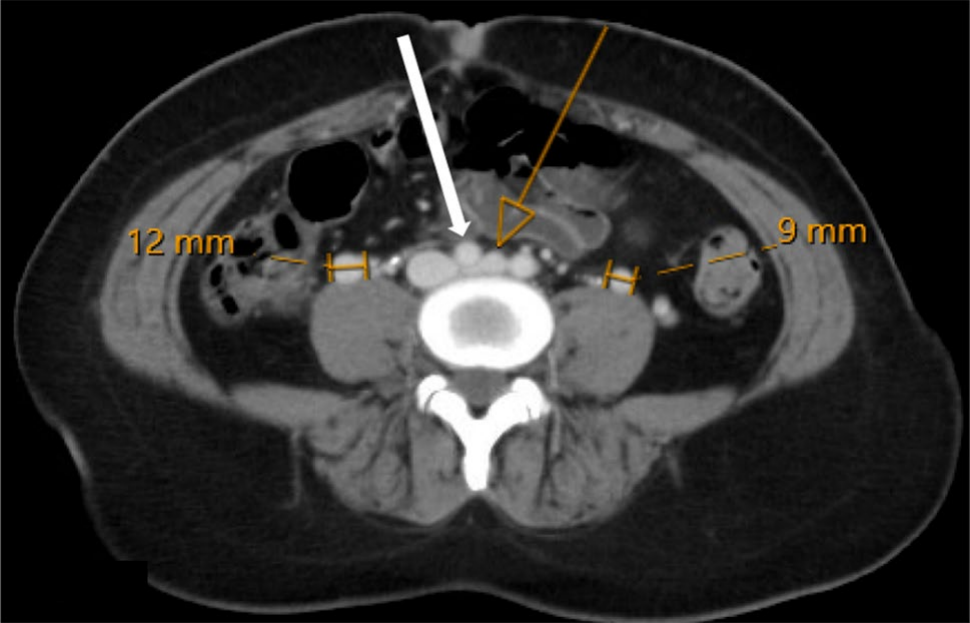
Iliac vein compression in a patient with pelvic venous disorder. 35-year-old multiparous female presenting with several months of pelvic pain and painful vulvar varicosities. Axial contrast-enhanced computed tomography shows focal narrowing of the left common iliac vein (orange arrow) by the right common iliac artery (white arrow) with associated dilated ovarian veins (left = 9 mm, right = 12 mm; orange measuring calipers)

Proximal VOO, defined as iliac vein stenosis/obstruction, including nonthrombotic iliac vein lesion (NIVL), and/or aortomesenteric left renal vein (LRV) compression, was ascertained using the highest-confidence evidence available: intravascular ultrasound/venography, then duplex ultrasound, then CT/MR report findings [15–18]. Representative examples of aortomesenteric LRV compression and iliac vein compression are shown in Figs. 4 and 5, respectively. Patients without any qualifying compression assessment were retained for GV analyses but excluded from compression analyses.

### Symptom Adjudication and Case/Control Definition

Two researchers reviewed emergency department, primary care, obstetrics/gynecology, and radiology documentation within ± 12 months of the index imaging date to classify symptom status. To mitigate under-reporting, researchers searched both structured fields and free-text notes (review of systems, problem lists, obstetrics/gynecology, interventional radiology, and primary care documentation). Charts with insufficient documentation were excluded rather than classified as asymptomatic; disagreements were resolved by consensus with a senior reviewer.

1. Cases (symptomatic PeVD): documentation of ≥ 1 PeVD-associated symptom (CPP, post-coital pain/dyspareunia, vulvar/perineal varices, LE swelling/varices attributed to pelvic reflux, or left flank pain/hematuria) either listed as the imaging indication or documented during evaluation.
2. Controls (asymptomatic): no documentation of PeVD-related symptoms (including explicit symptom denial when present) within the same window.

### Standardized Variable Definitions

Operational definitions, ascertainment sources, and abstraction windows are summarized in Supplementary Table 1, including pelvic varices (cohort entry), GV diameter, iliac NIVL and aortomesenteric LRV compression, superficial varicosities (LE and superficial pelvic), symptom status, and covariates. LE varices were defined per Clinical-Etiology-Anatomy-Pathophysiology (CEAP) classification [19]. For proximal VOO, we used descriptive anatomic terminology consistent with Symptoms-Varices-Pathophysiology (SVP) classification [15,20]. Eponyms (e.g., May–Thurner syndrome, nutcracker) were accepted only when used in radiology interpretations and mapped to the corresponding anatomic variables; historical clinical labels without imaging support were not used to define compression.

### Pelvic Venous Escape Pathways (Conceptual Framework)

“Escape points” were considered collateral routes by which pelvic reflux/venous hypertension decompresses into superficial venous beds (e.g., inguinal/round-ligament, obturator, pudendal/perineal, gluteal pathways) [21,22]. In this study, escape pathways were operationalized by documented superficial pelvic varicosities and/or LE varices within ± 12 months (see Multivariable models).

### Statistical Analysis

Two analysis populations were prespecified: (1) the full cohort (n = 200) for descriptive summaries and group comparisons; and (2) a modeling subset with proximal VOO ascertainable (n = 189). We did not employ individual or frequency matching because (1) we sought to estimate the effects of age and comorbid venous disease on symptoms; (2) matching on age or related factors risked overmatching given their correlation with exposures; and (3) the sample size allowed multivariable adjustment. We prespecified a core adjustment set and evaluated additional candidate covariates using a change-in-estimate criterion (≥ 10% change in the GV diameter coefficient) and clinical judgment.

**Descriptive Comparisons** Continuous variables are reported as mean ± standard deviation (or median [interquartile range, IQR] where noted) and compared using independent-samples t-tests (or Mann–Whitney U, as appropriate). Categorical variables are shown as n (%) and compared using Fisher’s exact or χ^2^ tests.

**Unadjusted (Crude) Associations** For each candidate predictor, we estimated crude odds ratios (ORs) and 95% confidence intervals (CIs) using single-predictor logistic regressions with symptomatic status as the outcome.

**Multivariable Models** We fit a logistic regression in the modeling subset (n = 189) with symptomatic status as the dependent variable. Age (per 10 years) was included a priori, and additional candidate covariates were retained if they changed the GV diameter coefficient by ≥ 10% and/or based on clinical judgment. A secondary model simultaneously entered iliac and LRV compression separately.

Superficial pelvic varicosities were evaluated descriptively but excluded from multivariable models because of potential differential documentation and under-ascertainment outside targeted venous evaluations, and because of conceptual overlap with LE varices as superficial “escape pathway” markers. [22]

**Model Specification and Diagnostics** Model assumptions and performance were assessed using Box–Tidwell testing for linearity, variance inflation factors (VIFs) for collinearity, Hosmer–Lemeshow (HL) for calibration, and AUC for discrimination; influence was evaluated using standard diagnostics (leverage, deviance residuals, Cook’s distance, and coefficient-change diagnostics) with a prespecified sensitivity analysis excluding observations flagged by ≥ 2 metrics.

**Pre-specified Sensitivity Analyses and Age Stratification** Sensitivity analyses included excluding parity, modeling iliac and LRV compression as separate predictors (point estimates were similar and GV diameter remained non-significant), and estimating continuity-corrected ORs for sparse cells. Results were compared with the primary model.

Because symptom phenotypes and venous manifestations vary by age, we prespecified age-stratified analyses (< 50/ ≥ 50 years), an *age* × *compression* interaction test, and alternative age parameterizations (continuous per 10 years vs categorical); results were compared across specifications.

All tests were two-sided, with statistical significance set at *P* < 0.05. Estimates are presented as ORs with 95% CIs; p-values are descriptive and not adjusted for multiplicity. Analyses were performed in IBM SPSS Statistics, version 30.0.0.0 (IBM Corp., Armonk, NY, USA).

## Results

A total of 200 patients (mean age 52.4 ± 17.0 years) with parauterine/pelvic varices met inclusion criteria (Fig. 1). Eighty-four (42.0%) had ≥ 1 chronic symptom consistent with PeVD (cases) and 116 (58.0%) were asymptomatic (controls).

Baseline characteristics are summarized in Table 1. Symptomatic patients were younger than asymptomatic patients (43.8 ± 13.6 vs 58.7 ± 16.6 years; *P* < 0.001). Tobacco use was less common among symptomatic patients (19.0% vs 35.3%; *P* = 0.012). Several comorbidities were more prevalent in asymptomatic patients, including hypertension (36.2% vs 11.9%; *P* < 0.001), hyperlipidemia (31.0% vs 15.5%; *P* = 0.013), and malignancy (40.5% vs 5.9%; *P* < 0.001). In contrast, migraine was more common in symptomatic patients (33.3% vs 12.1%; *P* < 0.001). Body mass index, gynecologic history (adenomyosis, endometriosis, fibroids, parity), and mental health diagnoses did not differ significantly between groups.

**Table 1:** Baseline demographics and clinical characteristics by symptom status

Baseline Characteristics	All (n = 200)	Asymptomatic (n = 116)	Symptomatic (n = 84)	OR (95% CI)	*P* value^*^
Age, years, mean ± SD	52.4 ± 17.0	58.7 ± 16.6	43.8 ± 13.6	0.94 (0.92–0.96)	** < 0.001**
*Race/ethnicity, n (%)*					*Overall exact ****P* = 0.0069**⁺
Non-Hispanic White	140 (70.0)	78 (67.2)	62 (73.8)		0.351
Hispanic or Latino	28 (14.0)	12 (10.3)	16 (19.0)		0.099
Black	22 (11.0)	20 (17.2)	2 (2.4)		** < 0.001**
Asian	6 (3.0)	4 (3.5)	2 (2.4)		1.000
Other (combined)	4 (2.0)	2 (1.7)	2 (2.4)		1.000
BMI, kg/m^2^, mean ± SD	24.3 ± 4.8	23.9 ± 4.6	24.7 ± 5.1	1.04 (0.98–1.09)	0.227
Tobacco use, n (%)	57 (28.5)	41 (35.3)	16 (19.0)	0.43 (0.22–0.84)	**0.012**
*Gynecologic history*					
Adenomyosis, n (%)	11 (5.5)	3 (2.6)	8 (9.5)	3.96 (1.02–15.42)	0.055
Endometriosis, n (%)	17 (8.5)	7 (6.0)	10 (11.9)	2.10 (0.77–5.78)	0.198
Fibroids, n (%)	39 (19.5)	23 (19.8)	16 (19.0)	0.95 (0.47–1.94)	1.000
Parity, median (IQR)ª	3.0 (2–4)	2.5 (2–4)	3.0 (2–4)	1.13 (0.95–1.35)	0.183
*Other Comorbidities*					
Hypertension, n (%)	52 (26.0)	42 (36.2)	10 (11.9)	0.24 (0.11–0.51)	** < 0.001**
Hypothyroidism, n (%)	45 (22.5)	30 (25.9)	15 (17.9)	0.62 (0.31–1.25)	0.229
Hyperlipidemia, n (%)	49 (24.5)	36 (31.0)	13 (15.5)	0.41 (0.20–0.83)	**0.013**
Malignancy, n (%)	52 (26.0)	47 (40.5)	5 (5.9)	0.09 (0.03–0.25)	** < 0.001**
Hemorrhoids, n (%)	59 (29.5)	35 (30.2)	24 (28.6)	0.93 (0.50–1.72)	0.876
Migraine, n (%)	42 (21.0)	14 (12.1)	28 (33.3)	3.64 (1.77–7.48)	** < 0.001**
Pain disorder, n (%)ᵇ	38 (19.0)	24 (20.7)	14 (16.7)	0.77 (0.37–1.59)	0.584
Depression, n (%)	74 (37.0)	42 (36.2)	32 (38.1)	1.08 (0.61–1.94)	0.882
Anxiety, n (%)	87 (43.5)	50 (43.1)	37 (44.0)	1.04 (0.59–1.83)	1.000

Significant values are formatted in bold

^*^Percentages calculated within symptom strata; *p* values by t-test (continuous) and Fisher’s exact or χ^2^ (categorical), as appropriate

⁺Overall race/ethnicity distribution: *p* = 0.0069 by Fisher–Freeman–Halton exact test (Monte-Carlo). Row *p*-values are exploratory and unadjusted

ªParity available in n = 196; 112/116 asymptomatic and 84/84 symptomatic patients

ᵇComposite of non-migraine pain disorders including fibromyalgia, chronic back/neck pain, trigeminal neuralgia, or chronic pain syndrome

Abbreviations: BMI Body mass index, CI Confidence interval, IQR Interquartile range, OR Odds ratio, SD Standard deviation

Imaging and venous findings are shown in Table 2. Mean GV diameter did not differ between groups (8.89 ± 2.29 mm symptomatic vs 9.43 ± 2.50 mm asymptomatic; *P* = 0.121), with a mean difference of − 0.54 mm (95% CI, − 1.21 to 0.13). Proximal VOO was assessable in 189 patients and not assessable in 11 (Fig. 1).

**Table 2:** Imaging and clinical venous findings by symptom status

Variable	All (n = 200)	Asymptomatic (n = 116)	Symptomatic (n = 84)	OR (95% CI)	*P* value^*^
*Deep pelvic imaging markers (CT/MR at index)⁺*					
Gonadal vein diameter, mm, mean ± SD	9.20 ± 2.42	9.43 ± 2.50	8.89 ± 2.29	0.91 (0.80–1.03)	0.121
Pelvic varices on imaging, n (%)	200 (100.0)	116 (100.0)	84 (100.0)	–	–
Any proximal VOO, n (%)^¶^	27 (14.3)	3 (2.6)	24 (32.9)	18.45 (5.31–64.15)	** < 0.001**
Iliac vein compression, n (%)^¶^	19 (10.1)	2 (1.7)	17 (23.3)	17.30 (3.86–77.52)	** < 0.001**
Left renal vein compression, n (%)^¶^	16 (8.5)	2 (1.7)	14 (19.2)	13.52 (2.97–61.51)	** < 0.001**
*Superficial varicosities*					
Lower extremity varices, n (%)ª	52 (26.0)	15 (12.9)	37 (44.0)	5.30 (2.65–10.60)	** < 0.001**
Superficial pelvic varices, n (%)ᵇ	26 (13.0)	2 (1.7)	24 (28.6)	22.60 (5.17–98.89)	** < 0.001**

Abbreviations: *CEAP* Clinical-Etiology-Anatomy-Pathophysiology, *CI* Confidence interval, *CT* Computed tomography, *GV* Gonadal vein, *GSV* Great saphenous vein, *LE* Lower-extremity, *MR* Magnetic resonance, *OR* Odds ratio, *SSV* Small saphenous vein, *TVUS* Transvaginal ultrasound, *VOO* Venous outflow obstruction

Significant values are formatted in bold

^*^Percentages calculated within symptom strata; *p* values by t-test (continuous) and Fisher’s exact or χ^2^ (categorical), as appropriate

⁺Imaging markers (pelvic varices, GV diameter) were abstracted from the index CT/MR (venous phase when available) interpreted by board-certified radiologists; TVUS/duplex ultrasound/venography used when unavailable

^¶^Proximal VOO was ascertained on highest-confidence available assessment: intravascular ultrasound/venography, then duplex ultrasound, then CT/MR imaging. On CT/MR, compression was based on the interpreting radiologist’s integrated assessment of focal narrowing with secondary signs. Proximal VOO was assessable in 189 patients (asymptomatic n = 116; symptomatic n = 73) and not assessable in 11

ªLE varices were coded present when CEAP C2 varicosities of the superficial system (GSV/SSV) were documented on clinical exam and/or duplex within ± 12 months of the index exam; findings limited to C1 telangiectasia/reticular veins were not coded as LE varices

ᵇSuperficial pelvic varicosities (vulvar, perineal, gluteal, or posterior-thigh) were recorded when explicitly documented on exam/duplex or described on CT/MR within ± 12 months of index imaging; total n = 199

Comorbid venous findings were substantially more common among symptomatic patients, including LE varices (44.0% vs 12.9%), superficial pelvic varices (28.6% vs 1.7%), any proximal VOO (32.9% vs 2.6%), iliac vein compression (23.3% vs 1.7%), and LRV compression (19.2% vs 1.7%) (all *P* < 0.001). Overall, any proximal VOO was present in 27/189 (14.3%), iliac compression in 19/189 (10.1%), and LRV compression in 16/189 (8.5%) (Table 2). Because superficial pelvic varicosities were likely differentially documented and conceptually overlap with LE varices as superficial escape-pathways, they were evaluated descriptively but excluded in adjusted models.

**Unadjusted Associations** Crude odds ratios are shown in Tables 2 and 3. GV diameter was not associated with symptom status (OR 0.91 per mm; 95% CI 0.80–1.03). In contrast, symptomatic status was associated with younger age (OR 0.55 per 10 years; 95% CI 0.44–0.67; *P* < 0.001), LE varices (OR 5.30; 95% CI 2.65–10.60; *P* < 0.001), superficial pelvic varices (OR 22.60; 95% CI 5.17–98.89; *P* < 0.001), and any proximal VOO (OR 18.45; 95% CI 5.31–64.15; *P* < 0.001).

**Table 3:** Factors associated with symptomatic pelvic venous disorder: crude and adjusted associations

Predictor	Crude OR (95% CI)	*P* value	Adjusted OR (95% CI), Model A^¶^	*P* value	Adjusted OR (95% CI), Model B^¶^	*P* value
Gonadal vein diameter (per mm)	–	–	0.97 (0.81–1.14)	0.686	0.98 (0.83–1.14)	0.770
Age (per 10 years)	0.55 (0.44–0.67)	** < 0.001**	0.50 (0.39–0.65)	** < 0.001**	0.50 (0.39–0.65)	** < 0.001**
Lower extremity varices⁺	5.30 (2.65–10.60)	** < 0.001**	9.80 (3.76–25.50)	** < 0.001**	9.86 (3.60–24.46)	** < 0.001**
Superficial pelvic varicosities⁺	22.60 (5.17–98.89)	** < 0.001**	–	–	–	–
Any iliac *or* LRV compression⁺	18.45 (5.31–64.15)	** < 0.001**	10.89 (2.66–44.55)	** < 0.001**	–	–
NIVL/iliac vein compression⁺	–	–	–	–	5.04 (0.87–29.25)	0.071
LRV compression⁺	–	–	–	–	6.63 (1.18–39.13)	**0.037**

*Multicollinearity:* assessed with VIF; all < 2

*Model calibration*: HL_Model A_ χ^2^(8) = 4.65 (P = 0.795); HL_Model B_ χ^2^(8) = 6.55 (*P* = 0.586)

*Influence-excluded sample (n* = *179; n* = *10 met influential criteria):* any compression perfectly separated cases and controls (22 vs 0); adjusted OR not estimable. Continuity-corrected (Haldane–Anscombe) crude OR_Model A_ 107.9 (95% CI 6.41–1816.3; Fisher’s exact *P* < 0.001); OR_Model B(NIVL)_ 72.9 (95% CI 4.05–1311; Fisher *P* < 0.001); and OR_Model B(LRV)_ 51.7 (95% CI 2.77–962; *P* < 0.001)

Abbreviations: *CI* Confidence interval, *GVD* Gonadal vein diameter, *HL* Hosmer–Lemeshow, *LE* Lower-extremity, *LRV* left renal vein, mm Millimeter, *NIVL* Nonthrombotic iliac vein lesion, *OR* Odds ratio, *VIF* Variance inflation factor, *χ*^2^ Chi-square

Significant values are formatted in bold

^¶^Model A (primary adjusted model) covariates: GVD, age, LE varices, any compression; Model B covariates: GVD, age, LE varices, iliac and LRV compressions entered separately

⁺Binary variable coded as present/absent

**Multivariable Models** (Table 3). In the primary adjusted model (Model A; Table 3), GV diameter remained non-significant (adjusted odds ratio [aOR] 0.97 per mm; 95% CI 0.81–1.14; *P* = 0.686). Younger age (aOR 0.50 per 10 years; 95% CI 0.39–0.65; *P* < 0.001), LE varices (aOR 9.80; 95% CI 3.76–25.50; *P* < 0.001), and any proximal VOO (aOR 10.89; 95% CI 2.66–44.55; *P* < 0.001) were independently associated with symptomatic status.

In the secondary model entering iliac and LRV compression separately (Model B; Table 3), GV diameter again was not associated with symptoms (aOR 0.98; 95% CI 0.83–1.14; *P* = 0.770). LRV compression remained significant (aOR 6.63; 95% CI 1.18–39.13; *P* = 0.037), whereas iliac compression did not reach significance (aOR 5.04; 95% CI 0.87–29.25; *P* = 0.071). Calibration was acceptable for both models (HL χ^2^(8) = 4.65, *P* = 0.795 for Model A; HL χ^2^(8) = 6.55, *P* = 0.586 for Model B). Discrimination was good (AUC 0.82, 95% CI 0.76–0.89). Adding GV diameter did not improve model performance (likelihood-ratio test *P* = 0.592; AUC 0.822 with vs 0.823 without GV diameter; Supplementary Fig. 1). Linearity of the logit was supported for both continuous terms (Box–Tidwell P_GVD_ = 0.645; P_age_ = 0.085), and collinearity was negligible (all VIF < 2).

**Age-stratified and Sensitivity Analyses** In prespecified analyses, proximal VOO remained associated with symptoms among women < 50 years (aOR 7.49; 95% CI 1.44–38.85; *P* = 0.017) and ≥ 50 years (aOR 21.80; 95% CI 2.20–216.11; *P* = 0.008), with no evidence of *age* × *compression* interaction (*P* = 0.497). Conclusions for GV diameter and lower-extremity varices were unchanged across strata (Supplementary Table 2).

In the influence-excluded analysis (n = 179; Table 3 footnote), proximal VOO perfectly separated cases and controls (22 vs 0), precluding finite adjusted estimates; the Haldane-Anscombe-corrected crude OR was 107.9 (95% CI 6.41–1816.3). Site-specific corrected crude ORs are provided in Supplementary Table 3.

## Discussion

In this retrospective case–control study of 200 patients with pelvic varices, GV diameter did not differ between symptomatic and asymptomatic patients and was not predictive of symptom presence after adjustment. In contrast, markers of comorbid venous disease, particularly LE varices and proximal VOO, including NIVL and/or aortomesenteric LRV compression, were strongly associated with symptomatic status. These results suggest that static GV caliber is a poor discriminator of clinically relevant PeVD, whereas system-level venous pathology and VOO better identify symptomatic disease.

Our findings align with prior work demonstrating limited diagnostic value of GV diameter thresholds in isolation [11,23]. Studies have shown that ovarian vein dilation is common on cross-sectional imaging in asymptomatic women and may reflect physiologic changes (e.g., parity) rather than pathologic reflux [23]. Likewise, prior investigations report only modest sensitivity/specificity when using diameter alone to infer reflux [11,24]. Taken together with our data, these findings reinforce that dilation can be an incidental imaging phenotype and should be interpreted in clinical context rather than used as a standalone criterion for PeVD.

The strong association between symptomatic status and LE varices, as well as proximal VOO, supports a multifactorial model in which symptoms relate more to venous hypertension, reflux physiology, and collateral/escape pathways than to vessel diameter alone [14,20,25,26]. Superficial varices may represent clinically observable “escape pathways” through which pelvic venous hypertension decompresses into superficial venous beds [22,26]. Although detailed mapping of pelvic escape routes was not the focus of this study, superficial pelvic varicosities may have been under-ascertained outside targeted venous evaluations. Therefore, LE varices were considered the more consistently captured superficial marker in this cohort. Their independent association with symptoms supports the concept that superficial venous manifestations may capture functionally important reflux/hypertension better than static GV caliber. [5,21,22,26,27]

Clinically, these findings caution against relying on GV diameter thresholds to diagnose PeVD among patients with physiologic vein enlargement. Such an approach risks over-attributing symptoms to incidental venous dilation and may miss symptomatic individuals whose GV size does not exceed arbitrary thresholds. A more comprehensive evaluation should incorporate symptom assessment alongside objective evidence of pelvic venous pathophysiology (e.g., reflux assessment when available), proximal venous anatomy/outflow obstruction, and superficial manifestations of pelvic venous hypertension. [12–14]

This study has limitations inherent to its retrospective design. Symptom status was adjudicated from clinical documentation and may be vulnerable to under-reporting; we mitigated this by reviewing multiple settings and structured/free-text notes (including documented symptom denial when present) and excluding charts with insufficient documentation. Controls were not matched, and residual confounding by factors correlated with age and healthcare utilization is possible despite prespecified adjustment and age-stratified analyses. Reflux/hemodynamic measures were not uniformly available. Proximal VOO was uncommon (overall 14%), yielding wide CIs and sensitivity to sparse cells in some secondary analyses, though associations were directionally consistent across robustness checks. Imaging measures represent a single time point and may vary with physiologic/technical factors.

Prospective studies integrating standardized symptom instruments with multimodal imaging and hemodynamic parameters (e.g., reflux duration/velocity, collateral mapping, and standardized criteria for outflow obstruction) are needed to refine diagnostic pathways. Evaluating whether GV size has prognostic value for treatment response, despite limited diagnostic discrimination, may also help clarify its clinical role.

## Conclusion

Among women with pelvic varices, GV diameter did not differentiate symptomatic from asymptomatic patients and provided no incremental predictive value beyond clinical and venous comorbidity markers. In contrast, LE varices and proximal outflow obstruction were strongly associated with symptoms. These findings support diagnostic strategies that prioritize hemodynamic/pathophysiologic evidence and systemic venous disease features over static GV caliber thresholds when evaluating suspected PeVD.

## Supplementary Information

Below is the link to the electronic supplementary material.

Supplementary file1 (DOCX 55 KB)

## Declarations

### Prior Presentation

Presented as an **oral presentation** at the **CIRSE Annual Congress**, Barcelona, Spain (September 13–17, 2025).
