# Supplementary material for: Compression and Superficial Varicosities Outperform Gonadal Vein Diameter in Differentiating Symptomatic from Asymptomatic Pelvic Venous Disorders: A Case–Control Study
Source: Cardiovasc Intervent Radiol. 2026 Jun 7;49(7):1268–77. doi: 10.1007/s00270-026-04493-5 (PMC13337957; doi:10.1007/s00270-026-04493-5)
Supplement: Supplementary file 1 — Supplementary file1 (DOCX 55 KB) [file 270_2026_4493_MOESM1_ESM.docx]

**Supplementary Material**

**Compression and superficial varicosities outperform gonadal vein diameter in differentiating symptomatic from asymptomatic pelvic venous disorders: a case–control study**

**Journal**: *CardioVascular and Interventional Radiology (CVIR)*

**Authors**:

1. Estefania Gonzales, MD MPH¹^*^
2. Victoria Nguyen, MD¹^*^
3. Victoria Risner, MD¹
4. Lourens du Pisanie, MD²
5. Nicole Keefe, MD²
6. Priya Mody, MD²
7. Marueen P Kohi, MD, FSIR, FCIRSE²
8. Gloria Salazar, MD, FSIR, FCIRSE, FAHA²

^*^Co-first authors

**Affiliations:**

¹ Department of Radiology, University of North Carolina School of Medicine, Chapel Hill, NC, USA
² Division of Vascular and Interventional Radiology, Department of Radiology, University of North Carolina School of Medicine, Chapel Hill, NC, USA

**Corresponding Author:**

Estefania Gonzales, MD MPH
Department of Radiology
University of North Carolina School of Medicine

Chapel Hill, NC 27599, USA
Email: Estefania_gonzales@med.unc.edu

**Supplementary Table 1.** Variable definitions and ascertainment hierarchy.

| Domain | Variable (coding) | Operational definition | Source | Notes |
| --- | --- | --- | --- | --- |
| Imaging (inclusion) | Pelvic/ parametrial varices⁺ | Parauterine/pelvic varices > 5 mm on the index imaging study. | CT/MR prioritized (highest-quality contrast-enhanced venous-phase exam). TVUS or DUS used only if CT/MR was unavailable. Symptom adjudication window anchored to the index imaging date (±12 mo). | Used for cohort entry only. |
| Imaging (exposure) | Gonadal vein (GV) diameter (continuous, mm) | Maximal caliber along GV course on axial CT/MR; if CT/MR absent, abstracted as reported maximal caliber on TVUS or DUS. | As above. | Two independent readers; mean used when both available. |
| Proximal outflow obstruction^*^ | Iliac vein compression (NIVL)⁺ | Focal common/external iliac vein narrowing in a characteristic segment ± pre-stenotic dilatation/collaterals; eponym accepted if used in radiology interpretation (e.g., “May–Thurner”). | *IVUS ± venography*: stenosis/area reduction reported. *DUS*: iliac outflow obstruction criteria reported. *CT/MR*: focal compression/stenosis ± collaterals described. | No de-novo stenosis measurements; used thresholds only when explicitly reported. |
|  | Left renal vein (LRV) compression⁺ | Aortomesenteric LRV narrowing with hilar dilatation/beak/collaterals or explicit diagnosis (“LRV compression/nutcracker”). | *Venography*: renocaval gradient ≥3 mmHg (if reported). *Renal Doppler/DUS*: PSV and/or diameter ratio across compressed vs hilar/proximal segment >4–5:1 (if reported). *CT/MR*: hilar-to-aortomesenteric diameter ratio >4–5:1 ± classic morphology (e.g., beak sign) described. | No re-measurement of angle/ratios/velocities by investigators. |
| Superficial varices^¶^ | Lower-extremity (LE) varices⁺ | CEAP C2: dilated, palpable subcutaneous varicose veins (GSV/SSV systems/tributaries) ≥ 3 mm upright on exam and/or DUS. | EHR (PMH/physical exam/clinical notes) and imaging reports. | Reflux parameters not required; CEAP C1 (telangiectasias/reticular < 3mm) not counted.^17 Lurie^ |
|  | Superficial pelvic varicosities⁺ | Vulvar, perineal, gluteal, or posterior-thigh varices explicitly documented on exam/DUS or CT/MR. | EHR (PMH/physical exam/clinical notes) and imaging reports. | Marker of pelvic escape pathways; CEAP classification not applicable. |
| Outcome | Symptom status⁺ | Symptomatic PeVD if ≥1 PeVD-associated symptom documented within ±12 months of index date (CPP ≥6 months or recurrent; dyspareunia/post-coital pain; vulvar/perineal varices; LE swelling/varices attributed to pelvic reflux; or left flank pain/hematuria); asymptomatic if none documented. | EHR (ED, primary care, OB/Gyn, radiology notes). | Two reviewers; disagreements adjudicated by senior reviewer. Charts with insufficient documentation were excluded (not classified as asymptomatic). |
| Covariates | Age (continuous, years) | Age at index imaging date. | EHR. | Modeled per 10 years; prespecified stratification (<50/≥50). |
|  | BMI (continuous, kg/m²)ª | Closest documented BMI to the index imaging date. | EHR (vitals/encounter data). | Nearest date selected if multiple. |
|  | Tobacco use⁺ª | Yes if current or former (“ever”) use documented; otherwise no. | EHR (social history/problem list/clinical notes). | Classified from clinician documentation; not independently re-verified. |
|  | Gynecologic conditions⁺ª | Adenomyosis, endometriosis, fibroids—present if documented in problem list, operative report, or pathology. | EHR (problem list, operative/pathology reports, notes). | Abstracted; not independently re-verified. |
|  | Other comorbidities⁺ª | HTN, hyperlipidemia, malignancy, migraine, hemorrhoids, CPD, depression, anxiety, thyroid disease—coded present if documented. | EHR (PMH/problem list/clinical notes). | Abstracted; not independently re-verified. |
| *Abbreviations:* BMI, body mass index; CEAP, Clinical–Etiological–Anatomical–Pathophysiological; CPP, chronic pelvic pain; CPD, chronic pain disorder; CT, computed tomography; DUS, duplex ultrasound; ED, emergency department; EHR, electronic health record; GSV, great saphenous vein; GV, gonadal vein; HTN, hypertension; IVUS, intravascular ultrasound; LE, lower extremity; LRV, left renal vein; MR/MRI, magnetic resonance (imaging); NIVL, nonthrombotic iliac vein lesion; PeVD, pelvic venous disorder(s); PMH, past medical history; PSV, peak systolic velocity; SSV, small saphenous vein; TVUS, transvaginal ultrasound. *Consensus references:* SVP classification/consensus; CEAP; Imaging diagnoses were accepted as documented by board-certified radiologists and were not re-evaluated by study investigators.  *Ascertainment windows:* Unless specified, windows were ±12 months from the index imaging date. When a finding was not explicitly documented, it was coded absent; indeterminate statements were coded missing.  ^*^Compression variables were coded missing only when no qualifying assessment (IVUS/venography, duplex, or CT/MR report evidence) was available.  ^¶^We acknowledge potential under-ascertainment of LE/superficial pelvic varices when not solicited in referral indications; this would bias results toward the null and is addressed in Limitations.  ⁺Binary variable coded as present/absent; each comorbidity, including gynecologic conditions, was binary.  ªReported in Table 1 only (not included in multivariable models). | | | | |

**Supplementary Table 2.** Age-stratified crude and adjusted associations (<50 vs ≥50) with symptomatic PeVD.

| Stratum | Predictor | Crude OR (95% CI) | *P* value | Adjusted OR^*^ (95% CI) | *P* value |  |
| --- | --- | --- | --- | --- | --- | --- |
| <50 | GV diameter (per mm), *n = 100* | 0.86  (0.71–1.04) | 0.120 | 0.87  (0.67–1.10) | 0.240 |  |
|  | LE varices *n = 100* | 13.89  (3.07–62.84) | **<0.001** | 12.16  (2.53–58.31) | **0.002** |  |
|  | Any iliac/renal compression, *n = 90* | 10.36  (2.24–47.94) | **<0.001** | 7.49  (1.44–38.85) | **0.017** |  |
| ≥50 | GV diameter, *n = 100* | 1.02  (0.86–1.22) | 0.787 | 1.05  (0.86 –1.28) | 0.619 |  |
|  | LE varices, *n = 100* | 4.17  (1.49–11.66) | **0.007** | 4.23  (1.37 –13.10) | **0.012** |  |
|  | Any iliac/renal compression, *n = 99* | 24.06  (2.63–220.12) | **0.001** | 21.80  (2.20–216.11) | **0.008** |  |
| *Denominators:* Vary by predictor due to variable availability (compression assessable subset).  *Model calibration:* HL χ²(8) = 9.33 (P = 0.315) for <50; HL χ²(8) = 7.00 (P = 0.536) for ≥50. | | | | | |  |
| ^*^Adjusted models were fit within each age stratum among patients with assessable proximal compression and included GV diameter (mm), LE varices, and any iliac/LRV compression. | | | | | |  |

**Supplementary Table 3.** Continuity-corrected crude ORs for iliac and LRV compression.

| Predictor | Crude OR (95% CI) | *P* | Continuity-corrected OR^*^ (95% CI) | *P* |
| --- | --- | --- | --- | --- |
| Any compression | 18.45 (5.31–64.15) | <0.001 | 103.9 (6.41–1814.3) | <0.001 |
| NIVL/iliac compression | 17.30 (3.86–77.52) | <0.001 | 40.3 (2.30–705.5) | <0.001 |
| LRV compression | 13.52 (2.97–61.50) | <0.001 | 35.4 (2.01–624.7) | <0.001 |
| ^*^Haldane–Anscombe correction for sparse cells. | | | | |


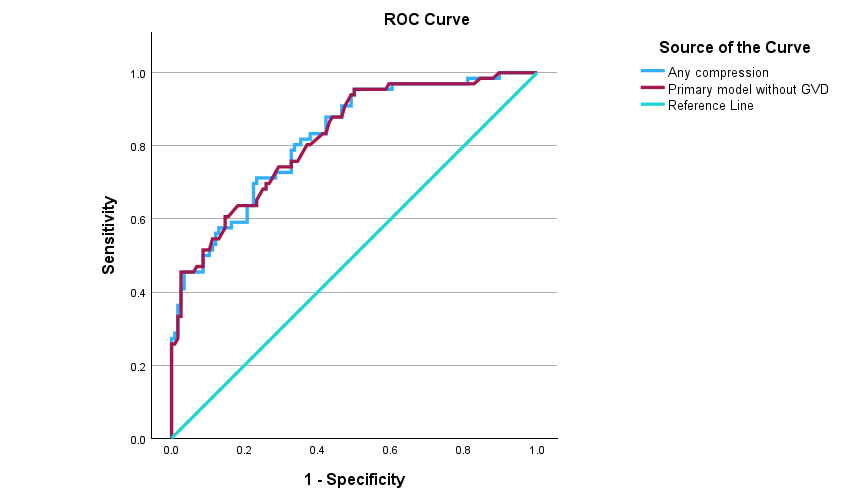


**Supplementary Figure 1.** ROC curves comparing the primary multivariable model with versus without gonadal vein diameter (complete cases, n=189). Discrimination was similar across models (AUC ≈0.82 for both), indicating no incremental predictive value of gonadal vein diameter beyond age, lower-extremity varices, and proximal outflow compression.
